# Therapy in the digital age: exploring in-person and virtual cognitive behavioural therapy

**DOI:** 10.1186/s12888-025-07063-0

**Published:** 2025-07-01

**Authors:** Julie Vizza, Sanaz Riahi, Olivia Jackson, Christen Potvin, David Rudoler

**Affiliations:** 1 Faculty of Health Sciences, Ontario Tech University, 2000 Simcoe St N, Oshawa, ON L1G 0C5 Canada; 2 Clinical Services, Practice & Chief Nursing Executive, Ontario Shores Centre for Mental Health Sciences, Whitby, ON Canada; 3 Department of Psychiatry, University of Toronto, Toronto, ON Canada; 4 Faculty of Health Sciences, University of Western Ontario, London, ON Canada; 5 Faculty of Social Sciences, University of Ottawa, Ottawa, ON Canada; 6 Population Health and Innovation in Mental Health, Ontario Shores Centre for Mental Health Sciences, Whitby, ON Canada

**Keywords:** Mental health, Mental health services, Cognitive behavioural therapy, Internet-based therapy, Client-centred care, Qualitative research, Thematic analysis

## Abstract

**Introduction:**

The adoption of client-centred care has become a foundational principle in mental health treatment, prioritising interventions tailored to the unique needs and preferences of clients across settings. Virtual or internet-based cognitive behavioural therapy (eCBT) has emerged as an effective, cost-efficient alternative to traditional, in-person CBT for a variety of mental health conditions, including anxiety and depression. Initially explored in experimental settings, eCBT gained substantial use during COVID-19, when the demand for accessible, remote mental health services were needed. Despite its broad implementation, limited research exists on the real-world experiences of clients who have participated in eCBT, particularly regarding its strengths and challenges compared to in-person therapy.

**Objectives:**

This study aimed to (1) explore the experiences of clients who have participated in both in-person CBT and eCBT, and (2) identify strengths and challenges associated with each modality from the client’s perspective.

**Methods:**

Clients were recruited from three outpatient clinics at Ontario Shores Centre for Mental Health Sciences in Whitby, Ontario. In-depth interviews were conducted with twelve clients between June and December 2023. Transcripts were analysed using Braun and Clarke’s six-step approach to thematic analysis.

**Results:**

Five main themes emerged from the data: (1) accessing therapy in a new way; (2) building a foundation for care: the client-provider relationship; (3) satisfaction with care; (4) addressing clients’ needs in the environment; and (5) client empowerment. Many clients expressed high satisfaction with eCBT, citing factors such as ease of access, flexibility, and the perceived effectiveness of virtual sessions in fostering mental health support. However, clients also noted challenges with technology, which could impact therapeutic engagement and the quality of the client-provider relationship.

**Discussion:**

The strengths and challenges identified in eCBT parallel those encountered in in-person settings, though eCBT was particularly appreciated by clients comfortable with digital environments. These findings emphasise the importance of client-centred care in virtual contexts, including the need for provider training in digital rapport-building and consideration of technological barriers. Ultimately, insights from this study can inform the refinement of eCBT delivery and support tailored approaches that align with the diverse needs of mental health service users in post-pandemic healthcare landscapes.

**Supplementary Information:**

The online version contains supplementary material available at 10.1186/s12888-025-07063-0.

## Introduction

Cognitive behavioural therapy (CBT) is a widely recognized and effective intervention for treating a range of mental health conditions, including anxiety, depression, and stress-related disorders [1]. Traditionally, CBT treatments have been delivered through in-person sessions, where the therapeutic meeting between a provider and client is central to the treatment’s success [2]. However, with increased use of digital health technology, virtual or internet-based CBT (eCBT) has emerged as an alternative mode of delivery. This virtual modality, which allows clients to participate in CBT through online platforms, gained popularity during the COVID-19 pandemic when access to in-person therapy was limited [3, 4]. eCBT is now being implemented more widely due to its potential to improve access, flexibility, and autonomy while maintaining the therapeutic efficacy of traditional in-person CBT [4].

Client-centred care, which emphasises tailoring interventions to client preferences and needs, is increasingly viewed as a cornerstone of effective mental health treatment [5]. As such, understanding client experiences with in-person and eCBT modalities is crucial for assessing care quality and identifying areas for improvement. Although there is a growing body of literature on the benefits of eCBT, including improved accessibility, cost-effectiveness, and flexibility [4, 6, 7], challenges related to the therapeutic relationships and technological barriers remain [7, 8].

The current study explores the experiences of clients who have participated in both in-person and eCBT to identify the strengths and challenges associated with each modality. For the purposes of this study, eCBT refers specifically to the virtual delivery of traditional CBT sessions, rather than app-based or asynchronous models of care. By focussing on client perspectives, this research seeks to contribute to the growing literature on eCBT, addressing gaps related to client experiences, and the transition from in-person to virtual care. Although previous studies have examined client experiences with eCBT, few have directly compared in-person and virtual CBT from the same clients’ perspectives. This study offers a unique contribution by drawing on the insights of individuals who have completed both modalities, enabling a within-person comparison of therapeutic processes, perceived effectiveness, and the relational dynamics of care. Furthermore, this study is particularly relevant in the context of the post-pandemic healthcare landscape, where hybrid and online treatment options are becoming important considerations of mental health service delivery. Findings from this research can inform clinical decision-making and service design by highlighting the conditions under which each modality may be more or less beneficial from the client’s point of view.

## Methods

### Study design

This qualitative study employed a reflective thematic analysis approach [9, 10] to explore client experiences with in-person CBT and eCBT. This approach was selected for its flexibility and its capacity to shed light on the complexities of lived experience in a nuanced and context-sensitive manner. In line with this approach, the analysis was interactive and interpretive, prioritising the researcher’s reflective engagement with the data over coded consensus. To ensure rigour, an audit trail documenting coding decisions and thematic development was maintained (JV), and reflective notes were kept by all coding research team members to examine how research assumptions and positionality shaped the analysis. This enhanced our ability to balance the participants’ lived experiences with our own critical reflections as researchers while providing in-depth and reflective portrayals of client experiences [10].

### Participant recruitment

Clients were recruited from outpatient clinics at Ontario Shores Centre for Mental Health Sciences (“Ontario Shores”) located in Whitby, Ontario, Canada. Ontario Shores is a publicly funded institution that offers specialised assessments and treatment options for individuals experiencing severe and complex mental health conditions [11]. A purposive sampling strategy was used to screen individuals who had experience with in-person CBT and eCBT. Eligible clients were adults aged 18 years and older who had completed sessions of each therapy modality within the past 24 months. Clients received the two modalities either sequentially or concurrently, with the order of delivery varying across individuals. Recruitment took place through direct outreach by clinic staff, by posting a recruitment flyer on the online client portal homepage (*My Health*,* My Way Patient Portal*), and through the institution’s already established Short-Messaging-Service (SMS) communication system.

### Data collection

Data were collected through semi-structured interviews with clients between June 2023 and February 2024. We determined that a sample of 12 participants offered sufficient diversity and depth of experience to address our research questions based on iterative reflection and preliminary analysis during data collection. This is in keeping with the interpretive ethos of reflexive thematic analysis [12]. Of note, in a study with a sample size of 12, the identification of themes and patterns is inherently more contextual and subjective than in studies with larger samples [13]. The terms ‘many’ and ‘some’ are not intended to make broad generalizations but to offer a clear understanding of the relative weight of a particular theme within this dataset.

Interviews were held via a secure video conferencing platform, *Zoom*. Each interview lasted between 25 and 60 min and was guided by a flexible interview protocol that covered key areas including clients’ perceptions of their therapeutic experiences, the perceived effectiveness of each modality, and the specific strengths and challenges they encountered. During the interview introduction, the interviewer clarified that the term ‘virtual eCBT’ referred to therapist-led sessions delivered via videoconferencing, to ensure clients understood the scope of discussion. The interview guide was developed by the research team based on relevant literature and practice knowledge and has not been previously published (see Supplementary File 1). Clients who participated in an interview were also offered a $25 dollar gift card as a token of the research team’s appreciation for sharing their time and expertise. All interviews were audio-recorded with clients’ consent and subsequently transcribed verbatim prior to analysis by a professional transcription service provider.

### Data analysis

Client interviews were analysed using Braun and Clarke’s six-step approach for reflective thematic analysis [9, 10]. This method allows for a nuanced exploration of patterns and themes across the data, facilitating a comprehensive understanding of clients’ experiences. The six steps included: (1) familiarization with the data, (2) generating initial codes, (3) searching for themes, (4) reviewing themes, (5) defining and naming themes, and, (6) producing the final report.

Three researchers (JV, OJ, CP), independently coded the data and discrepancies were resolved through discussion to ensure reliability. Themes were identified and organised around key topics related to the research objectives, with a focus on capturing the complexities of clients’ experiences in both in-person CBT and eCBT. A member of the research team (JV) was primarily responsible for the guardianship of transcription data and aggregation of the final analysis.

### Ethical considerations

A joint jurisdiction *Research Ethics Board Committee* provided ethical approval for this study (Ontario Shores Centre for Mental Health Sciences and Ontario Tech University, JREB #22-032-D). This study was not a clinical trial (Clinical trial number: not applicable). A member of the research team (JV) provided both oral and written information about the study to eligible clients with mental health conditions and obtained their written informed consent, providing them a copy of their consent for their records. Oral consent at the start of interviews was also audio recorded. Given the sensitive nature of clients’ experiences, the research team remained mindful of their potentially vulnerable positions and prioritised respect and dignity throughout the interview process and subsequent analysis [14]. In the event that a participant disclosed a risk of harm to themselves or others anytime during the interview, the interviewer (JV) had a protocol in place to immediately notify a senior member of the clinical care team and follow institutional procedures to ensure the client’s safety.

Clients were assigned identification numbers in all transcripts and reports, and any identifying information was removed during the transcription process. Additionally, steps were taken to ensure confidentiality and anonymity without compromising contextual information surrounding the phenomenon of interest [15].

## Results

A total of twelve (12) clients completed interviews. The sample was diverse in terms of self-reported age, gender, level of completed education, marital and employment status, and reason for seeking CBT/eCBT treatment, allowing for a broad exploration of client experiences across different demographics (see Table 1).

**Table 1:** Client demographics

Characteristic	*n* = 12
**Gender** ^**a**^	
Male	5 (41.6%)
Female	5 (41.6%)
Non-binary	1 (8.3%)
Prefer not to answer	1 (8.3%)
**Age (years)**	
18–25	1 (8.3%)
26–35	2 (16.6%)
36–45	3 (25%)
46–55	3 (25%)
55+	3 (25%)
**Education Level**	
High school or lower	4 (33.3%)
College/undergraduate degree	5 (41.6%)
Graduate degree or higher	3 (25%)
**Employment Status**	
Employed	5 (41.6%)
Unemployed	5 (41.6%)
Social assistance	1 (8.3%)
Student	1 (8.3%)
**Marital Status**	
Single	7 (58.3%)
Partnered	4 (33.3%)
Divorced/Separated	1 (8.3%)
**Self-Reported Mental Health Status**	
Excellent	0
Good	3 (25%)
Fair	5 (41.6%)
Poor	4 (33.3%)
**Self-Reported Diagnosis or Reason for Seeking Treatment** ^**b**^	
Anxiety	9 (75%)
Depression	9 (75%)
Post-traumatic stress disorder	2 (16.6%)
Other	9 (75%)

During analysis, five overarching themes were identified. These themes reflect clients’ experiences with both in-person CBT and eCBT, and provide insights into how these different modalities shape clients’ therapy journeys. The themes include: (1) accessing therapy in a new way; (2) building a foundation for care: the client-provider relationship; (3) satisfaction with care; (4) addressing clients’ needs in the environment; and (5) client empowerment. Each theme is explored in detail below. 

### Theme 1: accessing therapy in a ‘new way’

Clients identified that the shift to virtual therapy was different from traditional in-person service delivery. For many clients, the process of moving therapy online during the COVID-19 pandemic was unexpected and, in some cases, posed unanticipated challenges. For example, Client 1 described their abrupt shift to virtual therapy:

“… it was just, well, essentially, you know, one day you can go in and then the next day they’re like, OK, we’re closed. We’re not even in the office.” (Client 1).

This sudden transition led to a sense of uncertainty for some clients, particularly those who had never considered participating in virtual therapy. However, as they began to engage with eCBT, many found that the experience did not significantly differ from their in-person sessions. Client 4 shared their initial scepticism but eventual acceptance of the modality:

“It ended up being no different to me– the screen or the in-person. I mean at first, I thought I would prefer in-person. Um, I don’t know just a personal aspect of it, I guess. But once I did it, it was the same, so it didn’t matter. I would totally participate in virtual again.” (Client 4).

This quote underscores the adaptability of clients and the growing acceptance of virtual therapy as an alternative to traditional settings. Many clients noted that while they had initial concerns about the perceived impersonal nature of virtual sessions, their experiences were not negatively impacted by the non-traditional participation.

The convenience and accessibility of eCBT was also highlighted by several clients. For those who had difficulties attending in-person sessions due to geographical or logistical barriers, virtual therapy provided a more feasible solution. Clients emphasised that the flexibility to engage with therapy from the comfort of their own space was an important advantage, particularly for those with mobility issues or other commitments which made attending traditional sessions more difficult. Such was the case for Client 5, who was trying to juggle other necessary commitments such as education and employment:

“I was also in full-time college while I was doing that and working. So, like, as an adult, like, that was really, really hard to balance, because I need my education, I need to pay for rent, I have to work. So, the fact that I had that online therapy option,… it was just the accessibility and the comfort for me, personally, was just unprecedented against the experience that I had in the past of even just, like, taking the bus to that private [provider]….” (Client 5).

Overall, the accessibility and flexibility of eCBT addressed important barriers to care, enhancing client engagement and satisfaction. By overcoming logistical challenges and accommodating diverse client needs, virtual therapy not only replicates but, in some cases, surpasses the benefits of traditional in-person sessions. These findings underscore the importance of incorporating client-centred approaches into virtual care to ensure its continued success as a mainstream mental health service.

### Theme 2: Building a foundation for care: client-provider relationship

Establishing trust and rapport was a central concern for many clients, as they navigated the differences in interpersonal dynamics that come with participating in virtual therapy. While some clients noted the ease of building a connection with their therapist online, others expressed concerns about the limitations of virtual formats. Client 1 summarised this sentiment succinctly:

“I think the biggest thing is, I find there’s a limitation on nonverbal communication and cues through the computer.” (Client 1).

Client 1 highlights the important differences in the way relationships are built and communication is understood between clients and therapists. Some clients found the lack of physical presence, body language, and environmental cues in online settings hindered building strong relationships or extended the time required to establish connections. While others reported that eCBT effectively fostered therapeutic connections, with some even finding it easier to open up online. Client 5 shares their experience where the virtual modality did not impact their relationship with their provider:

“It wasn’t because it was online that I connected with my therapist, [therapist one], it was just because she was the right therapist for me. Versus, [therapist two], who was lovely, but I just didn’t have that connection. I don’t think it was because of online.” (Client 5).

The theme of developing a therapeutic foundation also encompasses the effort required on both sides– client and therapist– to bridge the gap created by the virtual space. Clients recognized that while initial sessions might feel less personal, the therapeutic relationship could still flourish with time and mutual effort.

### Theme 3: satisfaction with care

Clients’ satisfaction with eCBT was a prominent theme across interviews. Many clients reported a high level of satisfaction with the therapy they received, citing factors such as the quality of care, the alignment of the therapeutic approach with their needs, and the ease of access to therapy. Notably, most clients described the therapeutic content and structure across both modalities as consistent, and their overall sense of satisfaction was not dependent on modality but rather on contextual factors such as therapeutic rapport and session logistics. The overall perception of the effectiveness of the intervention, regardless of modality, was a significant factor in determining satisfaction. Some clients expressed satisfaction with the virtual format, noting that the ability to engage in sessions from their own environments allowed for greater self-reflection and comfort. Additionally, the incorporation of digital resources and interactive tools provided them with practical strategies to understand and manage their thought processes, which they could utilize independently after the sessions. As explained by Client 13 and Client 9:

“I also really appreciated the kind of information and knowledge to help me understand why my brain was working like that– something that helps to understand why it’s happening– and in the comfort of my own space. You know, that is really important for me. I can share that information with my wife or whatever and then we can discuss it when I feel an attack or whatever coming on. And I can revisit it later on too.” (Client 13).

and,

“One thing I will say that the [virtual] program did really well was they provided a workbook and we went through the workbook together online.… I really liked that because the workbook allowed us to have that resource. It had information that we worked through [online] in session, and we were able to have it outside of session too.” (Client 9).

These quotes demonstrate that virtual therapy achieved its primary purpose for some clients: providing mental health support, clarity on their conditions, and accessible resources. Clients appreciated that eCBT allowed them to continue receiving therapy when in-person sessions were not possible, contributing to their satisfaction, as noted by Client 12:

“You know in reality there’s not enough people to accommodate all the requirements for face-to-face [therapy]. So, this really is the best way for getting to people who need the help.” (Client 12).

Importantly, some clients also reflected on how behavioural experiments — a core component of CBT — were adapted into the virtual care they participated in. Client 9 described the added challenge of conducting exposures that required movement or relocation (e.g., handwashing) due to the constraints of a stationary device, and emphasized the importance of therapists engaging alongside clients to maintain a sense of accountability, presence, and support:

“I struggle with hand washing. So, when it’s like, oh yeah, go to the bathroom and do hand washing. Well, I can’t bring my computer with me to the bathroom. So, it’s kind of like that secret moment to be like, okay, I could probably fledge this and say I did this when I didn’t, right?… I think if the provider would kind of do it alongside the patient and like, kind of like take them to their bathroom as well and say, “hey, I’m in the bathroom with you”. Rather than it being like them at their desk and then the client in their bathroom.” (Client 9).

This sentiment highlights the importance of therapist modelling and creative adaptations in virtually delivered therapy. For this client, the therapeutic benefit of live, synchronous engagement, even in a virtual setting, was enhanced when the therapist matched their environment and co-participated in activities:

“I think like in person, you’re automatically in that space with that person but then virtually… it’s different than being in a physical space together. So even just like changing your environment to match theirs might help to show like, oh hey, we’re both in the bathroom.” (Client 9).

These insights echo concerns raised in the literature about maintaining active, experiential elements of CBT in virtual contexts. They also underscore the potential for eCBT delivery to offer unique opportunities for generalising therapeutic learning to real-world contexts, particularly when clients and therapists engage collaboratively and intentionally.

However, satisfaction with eCBT was also influenced by the quality of each client’s technological setup. For instance, clients with strong internet connections and updated devices reported smoother, uninterrupted therapy experiences, while those with weaker connections or older devices encountered frustration that impacted their engagement and satisfaction levels. Client 9 described difficulties with poor internet service in a remote area, sharing how frequent interruptions during emotional moments hinder the experience:

“I live in the middle of nowhere. So, the internet’s not great. So, a lot of internet cutting out on my end and the provider not being able to hear me correctly or vice versa… which also doesn’t help when you are kind of in the thick of it and you’re crying and they’re like, “Oh, yeah, can you, can you go back and repeat that?” or when they’re comforting you and giving you support and you can’t hear any of it.” (Client 9).

Similarly, Client 11 expressed frustration when a poor connection forced them to shift from a virtual session to a phone session, reducing the personal connection of the experience:

“Sometimes technology is not good. Once I was away from home at the time of my appointment, and I couldn’t get the reception and we were calling each other [online] and– I told her, “Call me on my phone”. She said, okay, we are not doing video because it’s not getting good reception. Honestly, I didn’t even want to meet after that.” (Client 11).

Clients were keen to voice suggestions for simplifying the technology to ease the therapy experience. Many also highlighted the complexity of navigating multiple technology layers, suggesting a streamlined approach that could benefit users:

“Really, what they need to do is help users set everything up so that we can just click one button and can avoid all the layers of technology, pages and such, to actually begin the work [laughs].” (Client 10).

These contrasts highlight the importance of addressing technological barriers to ensure equitable access to eCBT. Clients who experience interruptions or difficulty connecting to sessions report feeling disconnected and less supported, which can diminish their overall satisfaction with therapy. These findings emphasize that both technological readiness and the therapeutic adaptation, including active participation in CBT techniques, play crucial roles in client satisfaction and outcomes.

### Theme 4: addressing clients’ needs in the environment

Clients discussed how the settings of the therapy sessions, whether at home during the eCBT or within the walls of a clinical space during in-person therapy, impacted their ability to engage and benefit from the sessions.

Client 5 emphasised the role of the environment in shaping engagement:

“I would say the physical environment really can make a difference if I am engaged in a session or not. And even, like how much I want to talk to my therapist.” (Client 5).

For Client 5, the environment not only affected their willingness to engage with therapy but also influenced the depth of their participation. Some clients felt that being in their own space during eCBT sessions allowed for greater comfort and openness, while others felt that the lack of a designated therapy space at home could hinder their ability to remain focused. Such a perspective was shared by Client 11, who highlighted a particularly impactful experience with outdoor therapy:

“The environment creates physical stimulation too… when I went outside and I was doing it [eCBT session] there, I was in a green space, a beautiful place and not my home where all my depressing thoughts are staying.” (Client 5).

This example illustrates how the flexibility of eCBT enables clients to tailor their environments in ways that enhance their therapeutic experiences. However, while some clients appreciated the flexibility to tailor their environments for comfort and openness, others raised concerns about privacy and confidentiality.

For clients without a designated therapy space or those in shared living arrangements, the challenge of ensuring privacy during eCBT sessions sometimes became a barrier to fully engaging in therapy. Concerns about being overheard or lacking control over their environment undermined the sense of security typically felt in private, in-person therapy. Some clients, like Client 5, shared how this impacted their openness during therapy:

“I have my own designated room. But, like, yeah, I didn’t want my boyfriend to hear some of the OCD things I was talking about. And it’s not that I don’t trust him. It’s just that I don’t want him to hear them as I’m unpacking them, like, while I’m-I’m doing it. So, it was really important that I was in my room, and I knew that he was sleeping because he works overnight.” (Client 5).

Similarly, Client 9, who lived in a household with multiple family members, expressed related concerns:

“I live with my family, so I have four different people living with me. And like the walls aren’t thick, like it’s not that big of a house. So, privacy is kind of a concern… But I don’t know, I try not to focus on it when I have headphones in and the door closed.… I try to create a bubble of just me and the provider. But that’s definitely a huge, huge concern -- even though it’s family and, yeah, I love my family, but I don’t want them to know certain things, right?” (Client 9).

For Client 5 and Client 9, the physical proximity of others within the same home created a heightened concern about privacy. The clients’ ability to engage fully in therapy was closely tied to the level of control they had over their physical environment. Being overheard, even by a trusted partner or supportive family, posed a barrier to discussing deeply personal matters, indicating that some clients faced additional burdens when trying to create a therapeutic space at home.

Other clients, acknowledged the potential impact that worrying about privacy and confidentiality could pose but were not currently impacted by such factors:

“… the offices were nice, comfortable, private. So, as long as wherever you’re going for your help gives you that feeling, I don’t think it would be a difference.” (Client 4).

and,

“Throughout the years I have had different living situations. Living with roommates, my ex-wife, so, I have some of those complications. So, a [provider’s] office was probably better. Right now, though, I have none of those worries.” (Client 8).

These perspectives highlight how the physical environment of in-person therapy provides a sense of security that some clients find harder to replicate in virtual settings. The controlled and private nature of a provider’s office may contribute to clients’ sense of safety and openness, allowing them to focus on the therapy itself without the distractions or concerns related to privacy. Regardless of the setting, these experiences emphasise the importance of ensuring clients have access to an environment conducive to therapy, as not all clients may have the option to create such spaces.

### Theme 5: client empowerment

Clients highlighted how the therapy process fostered a sense of ownership over their mental health journey, enabling them to actively engage with strategies and tools tailored to their unique needs and circumstances. For example, during the interview with Client 5, it was acutely apparent that virtual therapy created a sense of empowerment:

“I really liked virtual therapy. I know that’s kind of a controversial thing with other people, but I was just really able to– there was something about just going from my mind to my electronic device that is so inherent in this day and age. I think that there was just an openness there that made speaking in that way a little bit easier.” (Client 5).

This reflection emphasises the sense of ease and control that some clients experience in a virtual therapeutic environment. eCBT gave clients the flexibility to engage with therapy in a way that was both comfortable and aligned with their personal needs and preferences. This convenience was raised by multiple clients, who noted that virtual therapy remained accessible even when physical or mental health challenges made attending in-person sessions difficult:

“But I can literally just wear my pajamas.… not to be, like, weird here. But I don’t have to put on a bra. I don’t have to do anything. I can do my OCD therapy in my bed if I want to. I’m having a really bad day. I don’t even want to be there. I would have cancelled otherwise.” (Client 5).

Another dimension of empowerment came from reduced barriers related to long wait times and unforeseen changes in personal circumstances:

“I got my diagnosis in 2020, and then I waited three years. I didn’t know I was going to be going to full-time school while I was on that waitlist. Like, just that wait time alone, is why I think giving an online option or having online therapy is good. Because you don’t always know what you’re going to be doing when you come up to the top of that waitlist.” (Client 5).

The practicality of virtual therapy was also appreciated for its convenience, especially in adverse conditions like winter weather or lack of transportation:

“Convenience. It’s extraordinarily convenient– being limited for transportation, getting to an [appointment] even in town.… but you know, in the middle of winter, it’s difficult, uh, bad traffic, it’s difficult. So, I mean, the convenience of this for some people would be extraordinarily helpful.” (Client 10).

Many clients noted that the flexibility and accessibility of eCBT allowed them to integrate therapeutic tools into their everyday lives. Client 3 reflected on the benefits of virtual therapy, particularly its alignment with their existing habits and routines:

“I actually really liked the strategies and tools we worked through online to reframe your mindset and to, uh, when you’re faced with a challenge to, um, reassess the situation and use those tools and knowledge to reframe your thinking. I mean, I already spend so much time online anyways– it makes sense.” (Client 3).

However, some clients felt that in-person sessions provided more robust access to additional resources, such as physical materials or library resources. Client 1 shared:

“One thing I do like about going to the office, I found a lot of the offices, they have resources there. Like usually they have a library so you can take books and access those resources. But you miss all of that if you’re at home.” (Client 1).

While virtual eCBT empowered clients through its accessibility and the ability to seamlessly integrate tools into daily routines, some clients still appreciated the tangible resources and physical environment offered in in-person settings.

## Discussion

This study explored client experiences with CBT and eCBT, identifying key themes that shed light on the nuances of transitioning from in-person therapy to participating in the use of virtual platforms/meeting spaces. The emerging themes contribute to our understanding of how clients navigate the transition between traditional and virtual therapy environments and highlight important considerations for the future of mental health service delivery.

### Adapting to virtual therapy

Previous research on eCBT has shown that while clients may initially feel uncertain about the effectiveness of online therapy, many reported positive outcomes after engaging with the modality [4, 9, 16]. The clients in this study echoed these findings, with several noting that eCBT was just as effective as in-person therapy once they were able to adapt to the new format. However, the transition to virtual therapy was not without challenges. For some, the sudden shift to online sessions during the COVID-19 pandemic disrupted established routines and created a sense of uncertainty. This finding aligns with existing research on the psychological impact of pandemic-related service disruptions [3, 4]. Despite these initial concerns, many clients highlighted the convenience and accessibility of eCBT, particularly for those with logistical barriers to attending in-person sessions. These advantages have been documented in the literature, with studies demonstrating that eCBT also increases access to care for individuals in rural or underserved areas [13, 17].

### Therapeutic relationships in virtual spaces

Clients in this study discussed the challenges of establishing rapport with their therapists in a virtual environment, particularly the lack of non-verbal cues and the physical presence that characterizes in-person therapy. This aligns with previous research suggesting that building a therapeutic alliance in virtual settings can be more difficult due to the absence of body language and the subtleties of face-to-face interaction [18].

Despite these challenges, many clients noted that strong therapeutic relationships could still be formed through eCBT, especially as clients and therapists adapted to the new modality. Some clients reported that virtual therapy made it easier for them to open up, as the physical distance created a sense of emotional safety. These findings echo the work of Berger (2017) [19], who found that some clients feel more comfortable sharing personal information in online therapy than in traditional settings, possibly because of the reduced immediacy and intensity of face-to-face interactions.

Nevertheless, the challenges of building rapport in virtual therapy underscore the importance of therapists adopting tailored approaches to foster client trust and connection in an online setting. While virtual modalities may filter out some nonverbal cues, these limitations can be addressed through intentional strategies such as active listening, frequent check-ins, and personalised follow-ups. Additionally, employing behavioural and technical adaptations, such as optimising video quality to enhance visual communication or using verbal affirmations to compensate for reduced nonverbal feedback, can further strengthen therapeutic connections in eCBT. These efforts are particularly crucial in ensuring that clients feel supported and engaged despite the physical distance inherent in virtual therapy [20, 21].

### Client satisfaction and treatment effectiveness

Satisfaction with eCBT was a central theme, with clients generally expressing positive evaluations, aligning with existing literature showing that eCBT is as effective as in-person CBT for a variety of mental health concerns [6]. Many clients were especially appreciative of the flexibility of eCBT, noting that the ability to engage in therapy from home or work made it easier to maintain consistent attendance.

A key factor influencing satisfaction was the quality of clients’ technological setups. Those with reliable internet access and updated devices reported smoother experiences, while clients with technological difficulties experienced frustration and disengagement. This finding highlights the importance of addressing the “digital divide” in mental health care, where individuals without access to technology may face barriers to receiving adequate care [13, 22].

### The role of the environment in therapeutic engagement

Clients in this study reported mixed experiences with the environmental factors surrounding eCBT. Some found that being in a familiar, comfortable environment facilitated openness and relaxation during sessions. Others, however, noted that distractions, noise, and lack of privacy at home made it difficult to fully engage in therapy. These findings are consistent with research showing that environmental factors can significantly influence the effectiveness of eCBT [23].

One novel aspect of this theme is the potential for eCBT to take place in more diverse environments, including outdoor settings, which some clients found therapeutic. This suggests that the flexibility of eCBT allows clients to tailor their therapy environment in ways that are not possible in traditional therapy. However, this flexibility also places additional responsibility on clients and providers to create a space that is conducive to therapy, which may pose additional challenges. To support clients in navigating these challenges, clients (and providers!) may benefit from discussing strategies to establish a conducive therapy environment [23, 24] and be provided resources to understand how a chosen setting may influence their engagement and therapeutic outcomes.

### Empowerment and autonomy in virtual therapeutic spaces

Many clients described how virtual therapy allowed them to take a more active role in managing their mental health, from choosing when and where to engage in therapy to feeling empowered by the knowledge and tools provided by their providers. Clients in this study also reported feeling empowered by the ability to share therapeutic insights with family members, a process that was facilitated by the flexibility of eCBT. This finding reflects the broader literature on the role of empowerment in eCBT, which enhances clients’ self-efficacy and control over their mental health [8, 25, 26].

However, empowerment was also closely tied to clients’ access to, and proficiency with technology. As noted in previous themes, clients who had reliable internet access and a perceived comfortable environment reported feeling more in control of their therapy, while those with technological difficulties felt disempowered. This finding suggests that while eCBT has the potential to enhance client autonomy, it also requires equitable access to the necessary tools and resources [8, 21, 25].

Interestingly, some clients described engaging in therapy from bed or while wearing pyjamas as empowering, reflecting their ability to choose a setting that felt comfortable or emotionally safe. While such flexibility may support engagement for some, it also introduces a potential tension between client-defined comfort and provider-defined readiness or professionalism, particularly in structured modalities like CBT [27].

### Clinical and policy implications

The findings have several implications for mental health practice and policy. By exploring client experiences across both in-person and virtual CBT, this study contributes a unique within-person perspective that helps illuminate the specific benefits and limitations of each modality as experienced by the same individuals. This offers more direct insight into how clients navigate differences in therapeutic process, engagement, and relational dynamics. These insights represent a perspective that is not commonly captured in existing literature.

First, the results suggest that virtual eCBT should be considered as a viable alternative to in-person therapy, particularly for clients who face barriers to accessing traditional services. However, implementation of eCBT must address digital inequities and ensure clients have access to technology and internet connectivity.

Second, clients’ emphasis on the importance of the therapeutic connection underscores the need for provider training on building relationships through digital platforms. This may involve strategic collaborations and developing skills in nonverbal communication and rapport building online [23, 28, 29].

Third, the role of the environment and shaping therapeutic experiences suggests providers should support clients to optimise their therapy setting. This includes guiding clients to create spaces conducive to effective eCBT sessions, establishing boundaries between therapy and daily life. Additionally, providers may require support and training to maintain the active components of CBT in virtual settings, including real-time behavioural experiments, therapist modelling, and use of flexible tools such as phone-based check-ins when appropriate. Ensuring eCBT remains experiential, rather than exclusively discussion-based, may help enhance its therapeutic impact.

Finally, eCBT’s potential to enhance client empowerment and autonomy should be leveraged to promote active engagement in treatment. Providers should consider incorporating additional resources and self-management tools to support client learning and skill development between sessions.

### Study considerations and future directions

While this study provides valuable insights into client experiences with in-person CBT and virtual eCBT, several considerations should be noted. The sample is drawn from a single healthcare institution, which may limit the transferability of the findings. Additionally, the study relied on retrospective accounts of therapy experiences, which may be subject to recall bias.

Along these lines, there was also variation in the order and timing of therapy modalities across clients, with some receiving in-person CBT prior to transitioning to eCBT. This may have influenced perceptions of therapeutic connection, particularly where rapport was first established in-person. Furthermore, the study did not formally explore potential interactions between clients’ presenting concerns, social circumstances (e.g., isolation, support systems), and their reported ability to connect with therapists during virtual sessions; however, these variables may have influenced the findings and represent important contextual considerations.

Future research should explore the long-term outcomes of eCBT versus in-person therapy, including the sustainability of therapeutic gains and impact on relapse rates. Longitudinal studies tracking outcomes across therapy modalities may provide a more comprehensive understanding of each approach’s strengths and limitations. Future work could also examine how individual client characteristics, such as presenting problems, mental health history, or social supports, interact with therapeutic modality to influence engagement and perceived connection. Investigating the experiences of diverse client populations could help to identify disparities in access and effectiveness of eCBT. Research on provider experiences and other client groups (e.g., varying digital literacy, diverse cultural or socioeconomic backgrounds, and different mental health conditions) would also complement the client perspective explored in this study.

## Conclusion

This study contributes to the growing body of literature on virtual therapy by exploring insights into clients’ experiences with eCBT. The findings demonstrate that while there are challenges associated with transitioning to virtual therapy, many clients find it to be a flexible, accessible, and empowering alternative to traditional in-person therapy. Specifically, the study highlights the significance of adapting therapeutic techniques to suit the digital environment, emphasizing the importance of establishing rapport and trust in virtual settings. These insights are important for providers as they navigate the evolving landscape of mental health care.

As eCBT becomes more integrated into mainstream therapeutic practices, ongoing evaluation and research will be essential for understanding its long-term effectiveness and client satisfaction. Ultimately, this research aims to inform best practices in the field, ensuring that eCBT not only meets the diverse needs of clients but also enhances their overall mental health and well-being.

## Electronic supplementary material

Below is the link to the electronic supplementary material.

Supplementary Material 1

## Data Availability

The datasets generated and/or analysed during the current study are not publicly available due to institutional policies and ethical considerations related to participant confidentiality. However, relevant anonymised excerpts from participant interviews are included within the manuscript to support the findings.

## Abbreviations

CBT: Cognitive behavioural therapy
eCBT: Virtual or internet-based cognitive behavioural therapy
