# Supplementary material for: Therapy in the digital age: exploring in-person and virtual cognitive behavioural therapy
Source: BMC Psychiatry. 2025 Jul 1;25:615. doi: 10.1186/s12888-025-07063-0 (PMC12211157; doi:10.1186/s12888-025-07063-0)
Supplement: Supplementary file 1 — Supplementary Material 1 [file 12888_2025_7063_MOESM1_ESM.pdf]

Ontario Shores
Centre for Mental Health Sciences

Ontario Tech University

CLIENTS - SEMI-STRUCTURED INTERVIEW GUIDE

Study title: Exploring client experiences with virtual cognitive behavioural therapy

Time	Instructions & Speaking Points
0:00 – 0:05	**<u>Welcome & Introductions</u>**<br><br>Hello,<br>**Provide a brief introduction.**<br><br>I will be talking to you today about your experiences participating in cognitive behavioural therapy (CBT) and about the different ways people can participate in CBT. For example, virtual or online cognitive behavioural therapy, which for the purposes of this study we are referring to electronic cognitive behavioural therapy (eCBT).<br><br>Because you have participated in CBT at Ontario Shores, you have been asked to participate in this study.<br><br>Thank you so much for agreeing to participate in this interview. I am looking forward to hearing about your experiences today. And so, with that we will get started.
0:05 – 0:15	**<u>Consent Form Review</u>**<br><br>Before our interview, you were provided with a copy of the consent form.<br><br>Did you have any questions about the information provided on the consent form? (*Answer any questions)*<br><br>Did you have any questions about the study? (*Answer any questions)*<br><br>Just to confirm, the interview will be audio recorded and I will also be taking notes during the interview. Do you have any questions about this?<br><br>Are you ready for me to begin the interview and start recording?<br><br>**Start recording.**<br><br>Just as a reminder, you can stop the interview at any time. You can choose to not answer a question for any reason. And you may withdraw your consent to participate at any time, without any negative consequences, and you do not need to give a reason for doing so.<br><br>Do you consent to take part in this study?

0:15– 0:25	**<u>Background & Context</u>**<br>1. I wonder if you could tell me a little about yourself, and what led you to seek cognitive behavioural therapy?<br>*Prompt: How has CBT treatment helped you?*<br><br>2. In your own words, what is cognitive behavioural therapy and how has it helped you?<br><br>3. So, I understand, you have participated in different delivery types of CBT, can you tell me a bit about that?<br>*Prompt: Why did you initially switch from one delivery method of CBT to another?*
0:25 – 0:40	**<u>CBT Therapy</u>**<br>4. Having had the experience of switching from one delivery method to another, do you have a preferred delivery method?<br>*Prompt: What are some of the things you like about [insert preferred modality].*<br>*Prompt: What are some of the differences between the different delivery types of CBT that you have participated in?*<br><br>4. During the COVID-19 pandemic, did you have any worries about not being able to participate in CBT?<br><br>5. Sometimes CBT can include learning a variety of skills and strategies – for example, muscle relaxation, deep breathing, and even, small homework assignments or tasks like journal writing or role playing – were you able to participate in these techniques virtually?<br>*Prompt if "no": Would you have been open to trying some of these techniques in virtual CBT sessions?*<br>*Prompt if "yes: Were they different than the skills and strategies you have participated in during in-person CBT sessions?*<br>Prompt if "yes": W*ere there any challenges because of the different mode of delivery of eCBT?*
0:40 – 0:70	**<u>Strengths & Challenges of Virtual CBT</u>**<br>6. What are some of the challenges you have experienced participating in CBT in a virtual environment?<br>*Prompt: Any concerns with your ability to access CBT services virtually?*<br>*Prompt: Have you experienced any challenges accessing or using the technology needed for your virtual CBT sessions?*<br><br>7. In your opinion, what are some of the benefits of attending virtual CBT sessions?<br>*Prompt: Has it made any difference in attending your CBT sessions?*<br><br>8. What keeps you motivated to continue virtual CBT sessions?<br>*Prompt: Did you ever consider not attending your sessions? If yes, what was the reason you considered not attending?*<br><br>9. Do you think the physical environment impacts your virtual CBT sessions?<br>*Prompt: Why or why not? (e.g., still participate in quiet setting, feel safe in providers presence, etc.)*

	10. Does your relationship with your provider play a role in the delivery of your virtual CBT treatment?<br>*Prompt: Could you tell me a little about that.*<br><br>11. Do you think virtual CBT can successfully be incorporated as a part of your care in the future?<br>*Prompt: Why or why not?*<br>*Prompt: What might that look like?*
0:75 – 0:80	**<u>Concluding Thoughts & Thank you</u>**<br><br>Well, we have talked about many aspects of your experiences participating in CBT and of your experiences participating in CBT virtually.<br><br>Is there anything you would like to add to our discussion, perhaps something you have just thought of or something we might not have discussed but you feel is important?<br><br>The study team's contact information is included on the information sheet and consent form you were provided. Please feel free to reach out with any additional information you think is important for us to know about your experiences or with any questions you have.<br><br>I am going to stop recording now and ask you a few demographic questions. Would that be okay?<br><br>**End Recording.**
0:80 – 0:85	**<u>Demographic Questions</u>**<br><br>So again, we hoped you might answer a few demographic questions to help provide some more context surrounding your experiences.<br><br>**Ask demographic questions and record answers in field notes.**<br><br>Just before we wrap up … Would you like to provide your email address to follow-up with any results that may be published from this study?<br><br>**If participant replies, "yes"** - Okay, great. What is the email address you would prefer to be contacted at?<br><br>**If participant replies, "no"** – That is completely okay.<br><br>And one final question, if it is okay with you, should any questions come up when I review our discussion, might it be alright to follow-up with you?<br><br>**If participant replies, "yes"** - That is wonderful. What method is best to contact you? Okay, well that concludes our interview. And again, I really appreciate you taking the time to chat with me today. Thank you.<br><br>**If participant replies, "no"** - that is alright, I completely understand. Okay, well that concludes our interview. And again, I really appreciate you taking the time to chat with me today. Thank you.
